# Familial clustering of dysbiotic oral and fecal microbiomes in juvenile dermatomyositis

**DOI:** 10.1038/s41598-024-60225-0

**Published:** 2024-07-12

**Authors:** Sean T. Koester, Albert Chow, Evan Pepper-Tunick, Peggy Lee, Mary Eckert, Laurie Brenchley, Pamela Gardner, Hyun Jung Song, Naisi Li, Adam Schiffenbauer, Rita Volochayev, Nastaran Bayat, Jeffrey S. McLean, Lisa G. Rider, Susan Shenoi, Anne M. Stevens, Neelendu Dey

**Affiliations:** 1 Translational Science and Therapeutics Division, Fred Hutchinson Cancer Center, Seattle, WA USA; 2 Department of Pediatrics, Division of Rheumatology, University of Washington, Seattle, WA USA; 3 Molecular Engineering and Sciences Institute, University of Washington, Seattle, WA USA; 4 School of Dentistry, University of Washington, Seattle, WA USA; 5 Center for Clinical and Translational Research, Seattle Children’s Research Institute, Seattle, WA USA; 6 Center for Immunity and Immunotherapies, Seattle Children’s Research Institute, Seattle, WA USA; 7 Office of the Clinical Director, NIDCR, National Institutes of Health, Bethesda, MD USA; 8 Environmental Autoimmunity Group, Clinical Research Branch, National Institute of Environmental Health Sciences, National Institutes of Health, Bethesda, MD USA; 9 Social and Scientific Systems, Inc., A DLH Holdings Corp. Company, Silver Spring, MD USA; 10 Department of Periodontics, University of Washington, Seattle, WA USA; 11 Department of Medicine, Division of Gastroenterology, University of Washington, Seattle, WA USA; 12 Microbiome Research Initiative, Fred Hutchinson Cancer Center, Seattle, WA USA; 13 Present Address: University of Kansas School of Medicine, Kansas City, USA; 14 Present Address: Loma Linda University, Loma Linda, USA; 15 Present Address: Oral Oncology at BC Cancer, Vancouver, BC Canada; 16 Present Address: Janssen, a Wholly Owned Subsidiary of Johnson & Johnson, Raritan, USA

**Keywords:** Paediatric rheumatic diseases, Microbiome

## Abstract

Juvenile dermatomyositis (JDM) is a rare immune-mediated disease of childhood with putative links to microbial exposures. In this multi-center, prospective, observational cohort study, we evaluated whether JDM is associated with discrete oral and gut microbiome signatures. We generated 16S rRNA sequencing data from fecal, saliva, supragingival, and subgingival plaque samples from JDM probands (*n* = 28). To control for genetic and environmental determinants of microbiome community structure, we also profiled microbiomes of unaffected family members (*n* = 27 siblings, *n* = 26 mothers, and *n* = 17 fathers). Sample type (oral-vs-fecal) and nuclear family unit were the predominant variables explaining variance in microbiome diversity, more so than having a diagnosis of JDM. The oral and gut microbiomes of JDM probands were more similar to their own unaffected siblings than they were to the microbiomes of other JDM probands. In a sibling-paired within-family analysis, several potentially immunomodulatory bacterial taxa were differentially abundant in the microbiomes of JDM probands compared to their unaffected siblings, including *Faecalibacterium* (gut) and *Streptococcus* (oral cavity). While microbiome features of JDM are often shared by unaffected family members, the loss or gain of specific fecal and oral bacteria may play a role in disease pathogenesis or be secondary to immune dysfunction in susceptible individuals.

## Introduction

Juvenile dermatomyositis (JDM) is a rare disease with increased morbidity and mortality that is characterized by stereotypical rashes and proximal muscle weakness, with an estimated incidence of 3.2 per million children in the United States^1^. While JDM primarily affects skin and muscle, it can also carry significant morbidity and mortality due to calcinosis and cardiac, respiratory, and gastrointestinal organ system involvement^2,3^. The etiology of JDM is thought to reflect the confluence of genetic predisposition, environmental exposures, and dysregulated immunity^4^. Immune-mediated diseases such as rheumatoid arthritis (RA), psoriasis, lupus, inflammatory bowel disease (IBD), and type 1 diabetes have been linked to alterations in fecal and oral microbiota^5–8^. Gingival inflammation is observed in 10–45% of JDM patients^9^, suggesting that plaque-induced gingival inflammation from oral bacteria may contribute to a systemic inflammatory response in JDM. However, there have been no studies investigating the role of the oral or gut microbiome in JDM patients. Identification of bacterial communities contributing to chronic inflammation in JDM could lead to an improved understanding of pathogenesis and offer new approaches for monitoring and treatment.

Disease cohort studies of the microbiome are statistically likely to identify differences based on the combination of multiple hypothesis testing and tremendous interpersonal microbiome differences^10^. In order to adjust for interpersonal differences and control for genetic predisposition to some extent, we studied children with JDM in comparison with their unaffected family members (siblings and parents) in this unique multicenter, prospective, observational cohort study. We reasoned that by controlling for shared family attributes, we would be able to identify microbiome signatures of JDM more clearly. This study design permitted (i) paired statistical analyses in which each JDM proband had familial controls and (ii) disease versus healthy cohort-based analyses.

## Results

### Clinical characteristics of study participants

We enrolled 28 children with JDM (mean ± SEM 10.0 ± 0.7 years of age, 46% female) and unaffected family members as controls (27 healthy siblings, 26 mothers, and 17 fathers; Table 1) across two study sites (Seattle Children’s Hospital [SCH], National Institutes of Health [NIH]). The JDM probands had mostly well-controlled disease activity with an average duration of disease of 4.25 ± 0.72 years; relevant clinical data are depicted in Table 1. Manual muscle test-8 (MMT-8) scores were available in 25 probands (89%) and ranged from 91 to 150. Based on these scores, 3 of 28 (11%) probands had abnormal muscle strength, most of which were in a range indicative of mild weakness (score greater than 135). Seven out of 28 probands had mild cutaneous disease activity (median score 2). Antinuclear antibodies (ANA) were recorded in 70% of JDM probands. Sixteen JDM probands were receiving one or more immunosuppressive medications, including methotrexate (*n* = 12), daily oral corticosteroids (*n* = 8), hydroxychloroquine (*n* = 7), intravenous immunoglobulin (IVIG) (*n* = 5), mycophenolate mofetil (*n* = 3), abatacept (*n* = 2), and/or tacrolimus (*n* = 1). Of the remaining probands, 10 were not receiving medical therapy for JDM, and 2 did not provide medication history.

**Table 1:** Summary of clinical and demographic characteristics of study participants.

	JDM probands (*n* = 28)	Siblings (*n* = 27)	Parents (*n* = 43)
Age, years	10.0 ± 0.7	11.3 ± 0.9	41.4 ± 1.3
Gender, female	13 (46%)	11 (41%)	26 (60%)
Race*			
Caucasian	20 (72%)		
Mixed race	4 (14%)		
Not reported	4 (14%)		
Selected labs and disease activity scores	Median (IQR)		
MMT-8 (maximum score 150)	150 (144–150)		
CDASI activity score (maximum score 96)	2 (0–5)		
CDASI damage score (maximum score 32)	0.5 (0–2)		
CRP (normal range < 5 mg/dl)	0.8 (0.2–1)		
ESR (normal range < 20 mm/hr)	7 (7–8)		
Aldolase (normal range < 7 U/L)	5.8 (4–7.8)		
CK (normal range 35–230 U/L)	118 (90–159)		
LDH (normal range 370–840 U/L)	606 (557–678)		
AST (normal range 5–41 U/L)	32.5 (23–36)		
ALT (normal range 5–30 U/L)	18.5 (13.75–28.5)		
Autoantibodies**			
Non-specific autoantibodies			
ANA (*n* = 24)	17 (70%)		
Anti-dsDNA antibody (*n* = 17)	1 (6%)		
Myositis-specific autoantibodies (MSA)			
Anti-MJ (NXP2) antibody (*n* = 8)	3 (38%)		
Anti-p155 (TIF1) antibody (*n* = 8)	2 (25%)		
Anti-MDA5 antibody (*n* = 8)	2 (25%)		
Anti-Mi2 antibody (n = 23)	1 (4%)		
Myositis-associated autoantibodies (MAA)			
Anti-Ro antibody (*n* = 23)	3 (13%)		
Anti-U1RNP (*n* = 24)	2 (8%)		
Anti-U2RNP (n = 24)	1 (4%)		
Negative all autoantibodies (n = 8)	1 (13%)		
Negative MSA/MAA (n = 8)	4 (50%)		
Medications***			
Methotrexate	12 (46%)		
Oral corticosteroids (i.e., prednisone)	8 (31%)		
Hydroxychloroquine	7 (27%)		
Intravenous immunoglobulin G (IVIG)	5 (19%)		
Mycophenolate mofetil	3 (12%)		
Abatacept	2 (8%)		
Tacrolimus	1 (4%)		
None	5 (19%)		

*Race and ethnicity were self-reported. Mixed race denotes combinations of Caucasian, Native American, Alaskan Native, Black/African American, Native Hawaiian, and/or Pacific Islander.

**Out of 28 JDM probands, 8 had all 19 myositis autoantibodies assessed in the serum, 2 had no autoantibodies assessed, and the remainder had between 3 and 15 autoantibodies assessed. The numbers of JDM probands who had a given antibody checked are shown in brackets following each antibody name. The following antibodies are not included here because no JDM probands were positive for them: anti-Jo1, anti-PL-7, anti-PL-12, anti-EJ, anti-OJ, anti-KS, anti-SRP, anti-PM-Scl, and anti-Ku.

***No medication data were available for 2 JDM probands; therefore, percentages reflect a denominator of 26. Total percentages do not add to 100% because several JDM probands were receiving multiple medications.

### Oral and fecal microbiomes clustered by family unit and particularly by sibling relationship

**Figure 1:**
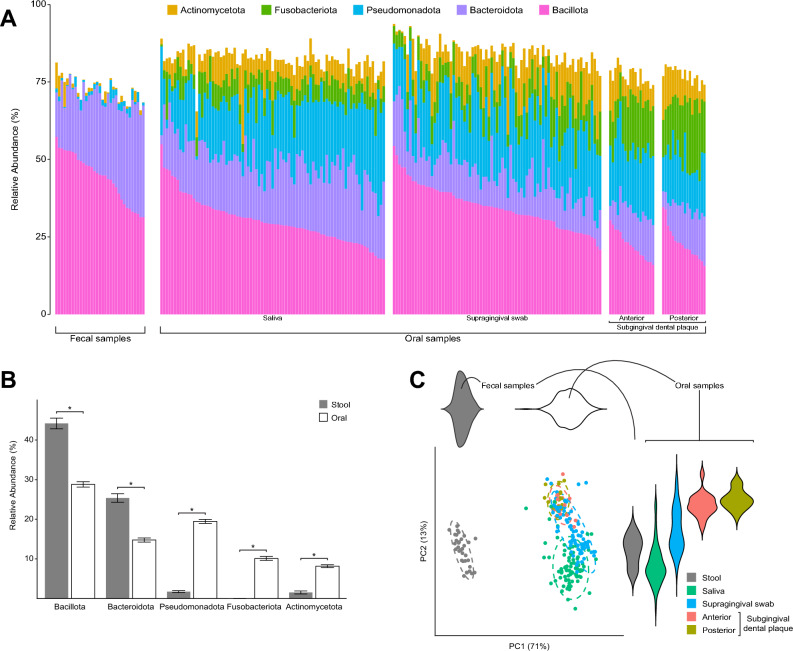
(**A**) Microbiota community structure at the phylum level of all samples sequenced in this study, separated by sample type. The 5 most prevalent phyla are represented. (**B**) Phylum-level differences of stool and oral samples. (**C**) PcoA plot based on weighted UniFrac distances of all samples in this study. The greatest separation is seen between fecal and oral samples along PC1, which accounts for the greatest variability throughout the dataset. Different oral sample types separate along PC2.

To identify key differences in microbiome diversity and community structure in JDM, we performed 16S rRNA amplicon sequencing of the V4 hypervariable region of oral and gut samples collected from these family-based cohorts (Tables S1, S2A). After filtering, we observed a total of 3,304 amplicon sequence variants (ASVs) throughout our dataset (Table S2B), of which 1,448 ASVs were detected in gut microbiomes, 1,928 ASVs were detected in oral microbiomes, and 72 ASVs were seen in subsets of both fecal and oral gut microbiomes (Fig. S1). *Bacillota* ﻿(formerly known as *Firmicutes*) and *Bacteroidota* (formerly known as *Bacteroides*) predominated in the fecal microbiomes (*p* < 10^–10^, two-tailed Student’s *t*-test), whereas *Pseudomonadota* (formerly known as *Proteobacteria*), *Fusobacteriota* (formerly known as *Fusobacteria*), and *Actinomycetota* (formerly known as *Actinobacteria*) were also highly prevalent in the oral microbiomes (*p* < 10^–21^, two-tailed Student’s *t*-test; Fig. 1A,B). Principal coordinates analysis (PCoA) based on weighted UniFrac distances demonstrated stark differences in oral and fecal microbiota community structures (Fig. 1C). Matching expectations of greater diversity and biomass in fecal than in oral samples, fecal microbiomes were characterized by greater alpha diversity than oral microbiomes (*p* < 10^–13^, two-tailed Student’s *t*-test; Fig. S2A); similarly, genomic DNA concentrations were significantly higher in fecal than oral samples (*p* < 10^–11^, two-tailed Student’s *t*-test; Fig. S2B).

We then calculated microbiome similarities within and between family units using UniFrac, a metric for quantifying pairwise phylogenetic distances between samples. Family unit was the predominant factor explaining variance in both weighted and unweighted UniFrac distances between samples for each sample type (*p* < 0.001 in fecal, saliva, supragingival swab, and posterior subgingival dental plaque samples; *p* < 0.01 in anterior subgingival dental plaque samples, permutational multivariate analysis of variance [PERMANOVA]).

**Figure 2:**
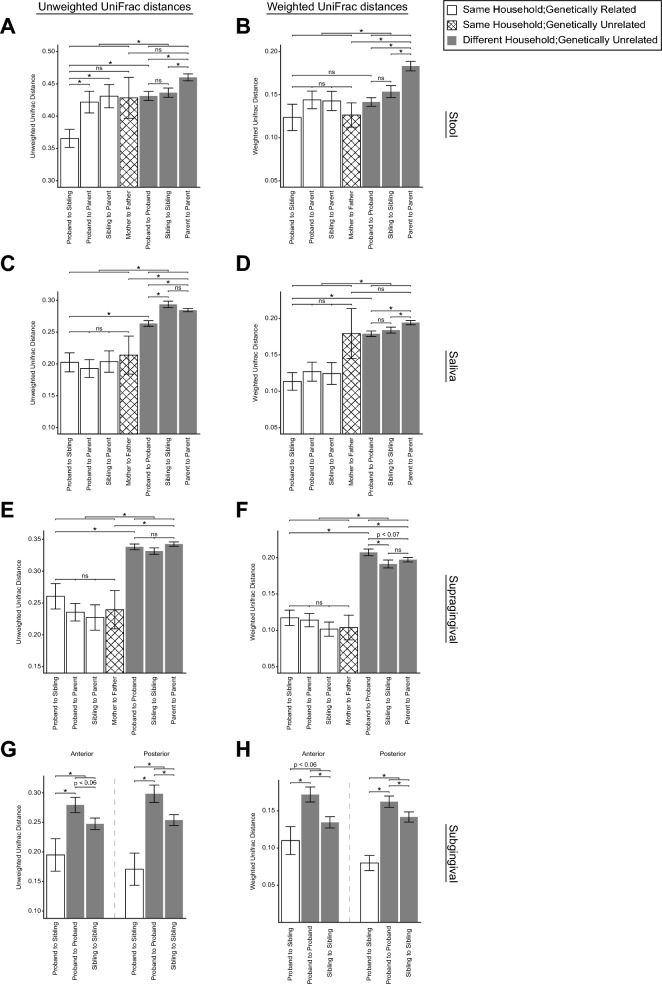
UniFrac distances between (**A**,**B**) fecal, (**C**,**D**) salivary, (**E**,**F**) supragingival swab samples, and (**G**,**H**) anterior/posterior subgingival dental plaque microbiomes of different cohorts in our study. Both unweighted (**A**,**C**,**E**,**G**) and weighted (**B**,**D**,**F**,**H**) UniFrac distances are shown. *p < 0.05.

Paired unweighted UniFrac distances between JDM probands and their own siblings were significantly smaller than the paired distances between JDM probands and their own parents (*p* < 0.05, two-tailed Student’s *t*-test; Fig. 2A). In fact, the unweighted UniFrac distances between siblings was significantly smaller than the distances between JDM probands in our cohort (*p* < 0.002, two-tailed Student’s *t*-test). This trend was also seen in a comparison of weighted UniFrac distances (*p* < 0.08, two-tailed Student’s *t*-test; Fig. 2B). In other words, siblinghood was the most reliable of three indicators of fecal microbiome similarity in our study, which also included family unit and JDM diagnosis.

Saliva, supragingival swab, and subgingival dental plaque microbiomes were highly similar between members of families (Fig. 2C–H). The UniFrac distances between siblings’ oral microbiomes were not significantly different from the distances between JDM probands and their own parents (or between the healthy siblings and their own parents; two-tailed Student’s *t*-tests; Fig. 2C–G). *Intra*-familial distances were significantly smaller than all *inter*-familial distances (*p* < 0.005, two-tailed Student’s *t*-test; Fig. 2C–H). As with gut microbiomes, the UniFrac distances between oral microbiomes of JDM probands and their healthy siblings were significantly less than the distances between all JDM probands (*p* < 0.02, two-tailed Student’s *t*-test), further supporting the notion of a stronger effect of sibling relationship than JDM diagnosis. UniFrac distances between oral microbiomes of parents within a household were significantly smaller than the distances between adults in different households (*p* < 0.05 for all comparisons, except weighted UniFrac distances of saliva samples, two-tailed Student’s *t*-test; Fig. 2C–F)—a trend seen in fecal microbiomes as well (Fig. 2A).

Alpha diversity was significantly lower in children compared to adults (271.4 ± 8.2 vs 308.5 ± 10.9 [observed species]; 288.0 ± 8.3 vs 325.1 ± 11.9 [Chao1]; *p* < 0.02, two-tailed Student’s *t*-test). Further evidencing sibling-associated microbiome similarities, alpha diversity was not significantly different in a paired analysis comparing JDM probands to their unaffected siblings (*p* > 0.05, paired two-tailed Student’s *t*-test; both observed species and Chao1 metrics).

### Family units of JDM probands exhibit dysbiotic microbiomes

**Figure 3:**
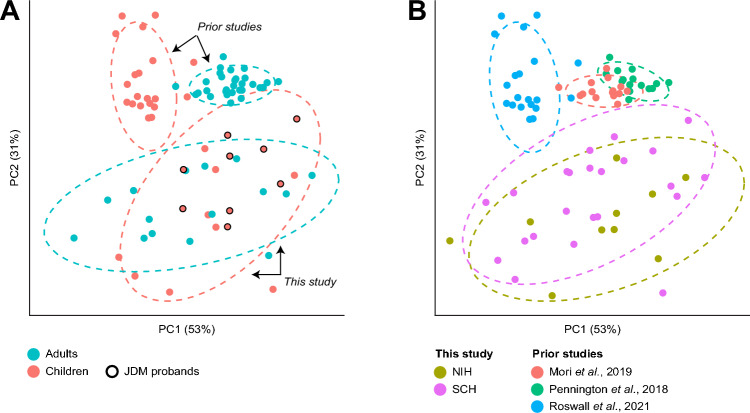
(**A**) PcoA plot based on weighted UniFrac distances between stool samples in our study and from 3 other studies employing the same sequencing strategy, color-coded by age group (children vs adults). (**B**) Same PcoA plot as in (**A**), color-coded by study site.

We next asked whether the familial clustering seen in our cohort of JDM probands and their nuclear families reflected familial dysbiosis. To compare gut microbiomes with healthy individuals lacking any known genetic or familial predisposition to JDM, we performed a cross-dataset analysis of adults^11,12^ and children (selecting data from children ≥ 3 years of age)^13^ whose gut microbiomes had been previously profiled by other groups using the same sequencing strategy and otherwise similar methods (including use of the same sample collection kit in one study^11^; Table S2C). To mitigate potential biases imparted by batch effects, we employed Conditional Quantile Regression (ConQuR) to first remove microbiome batch effects^14^. Stool samples from individuals in our study (children with JDM, their unaffected siblings, and/or their parents) all clustered separately from samples from these three published datasets (*p* < 0.001 for both weighted and unweighted UniFrac distances, PERMANOVA; Fig. 3A,B). Acknowledging that there nonetheless remain differences in specific populations, protocols, and reagents that cannot be entirely controlled for, this may suggest that microbiomes of not only JDM probands, but entire JDM family units, are markedly different from previously reported healthy microbiomes.

### Altered representation of immunomodulatory taxa in JDM probands

JDM was a significant factor explaining variance in weighted UniFrac distances in fecal and supragingival swab samples (*p* < 0.03, PERMANOVA). Therefore, we next sought to identify specific bacterial taxa differentiating children with JDM from their unaffected siblings in all fecal and oral samples, reasoning that differences in specific individual taxa may be biologically important. Our unique study design permitted us to adjust for microbiome biases attributable to family and age, e.g. via paired comparisons of JDM probands to their siblings. In this section, we calculate significance without adjusting for multiple comparisons, a necessity based on small sample sizes given the rarity of JDM in the population. Thus, we refrain from reporting *p*-values or *q*-values, instead describing distributions of taxonomic abundances in different cohorts.

**Figure 4:**
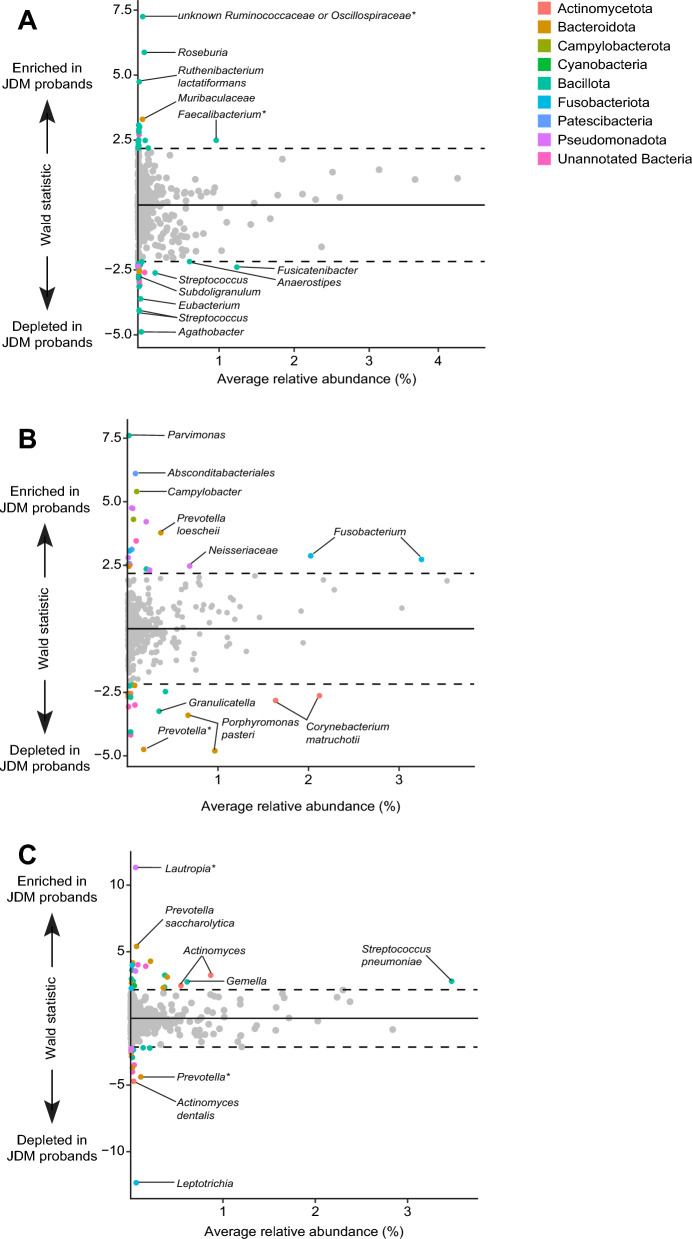
Differentially abundant ASVs in (**A**) fecal microbiomes, (**B**) anterior subgingival dental plaque samples, (**C**) and posterior subgingival dental plaque samples of JDM probands compared to unaffected siblings. As described in the text, post-hoc taxonomy was assigned based on BLAST results to the 4 ASVs designated by an asterisk (*) that were initially classified as unannotated bacteria.

Approximately 4% of bacterial ASVs were differentially abundant in fecal microbiomes between JDM probands and their siblings (Fig. 4A). Most of these ASVs were rare *Bacillota*, namely *Clostridia* and *Bacilli*. Two ASVs significantly enriched in the stool of JDM probands compared to their healthy siblings were classified as unannotated bacteria. An NCBI BLAST search of these sequences suggested that these reads represent an unknown *Ruminococcaceae* or *Oscillospiraceae* species (0.08 ± 0.03% JDM vs 0.05 ± 0.03% siblings) and an uncultured *Faecalibacterium* (which is in the *Oscillospiraceae* family; 1.13 ± 0.15% JDM vs 0.95 ± 0.12% siblings). Additionally, an unknown *Roseburia* species (0.15 ± 0.05% JDM vs 0.02 ± 0.02% siblings) and *Ruthenibacterium lactatiformans* (0.03 ± 0.007% JDM vs 0.007 ± 0.005% siblings) were significantly enriched in the stool of JDM probands. In contrast, unknown species of the genera *Agathobacter* (0.02 ± 0.02% JDM vs 0.07 ± 0.04% siblings), *Anaerostipes* (0.55 ± 0.13% JDM vs 0.83 ± 0.25% siblings), *Fusicatenibacter* (0.9 ± 0.2% JDM vs 1.7 ± 0.3% siblings), *Streptococcus* (3 ASVs: 0.1 ± 0.05% JDM vs 0.4 ± 0.2% siblings, 0.007 ± 0.008% JDM vs 0.02 ± 0.01% siblings, and 0.007 ± 0.004% JDM vs 0.02 ± 0.02% siblings)*,* and *Subdoligranulum* (0.0004 ± 0.0004% JDM vs 0.01 ± 0.01% siblings) were significantly depleted in JDM probands.

A greater proportion and diversity of ASVs were differentially abundant in the oral microbiomes between JDM probands and their siblings. Approximately 8% of ASVs identified in the anterior subgingival dental plaque were significantly different between JDM probands and their healthy siblings (Fig. 4B). The most enriched ASV in JDM probands was an unknown *Parvimonas* species (0.03 ± 0.03% JDM vs 0.006 ± 0.006% siblings). Meanwhile, *Corynebacterium matruchotii* (2 ASVs: 1.9 ± 0.3% JDM vs 2.4 ± 0.1% siblings and 1.4 ± 0.2% JDM vs 1.8 ± 0.08% siblings), *Porphyromonas pasteri* (2 ASVs: 0.95 ± 0.1% JDM vs 0.1 ± 0.1% siblings and 0.6 ± 0.1% JDM vs 0.7 ± 0.1% siblings), *Granulicatella* (0.3 ± 0.03% JDM vs 0.4 ± 0.04% siblings), *Prevotella loescheii* (0.3 ± 0.1% JDM vs 0.4 ± 0.2% siblings), and an unclassified species with high sequence identity to *Prevotella* (0.1 ± 0.07% JDM vs 0.3 ± 0.1% siblings) were significantly depleted in JDM probands.

In posterior subgingival dental plaque samples, approximately 7% of identified ASVs were significantly differentially abundant between JDM probands and their siblings (Fig. 4C). ASVs representing *Actinomyces* species (2 ASVs: 1.0 ± 0.2% JDM vs 0.8 ± 0.1% siblings and 0.6 ± 0.1% JDM vs 0.5 ± 0.07% siblings), *Gemella* (0.7 ± 0.1% JDM vs 0.6 ± 0.1% siblings), *Streptococcus neumonia* (3.6 ± 0.5% JDM vs 3.3 ± 0.4% siblings), and an unclassified ASV with high sequence identity to *Lautropia* (0.08 ± 0.08% JDM vs 0.03 ± 0.03% siblings) were enriched in JDM probands. Additionally, *Actinomyces dentalis* (0.01 ± 0.01% JDM vs 0.05 ± 0.01% siblings), *Leptotrichia* (0.03 ± 0.03% JDM vs 0.09 ± 0.09% siblings), and an unclassified ASV with high identity to *Prevotella* (0.05 ± 0.03% JDM vs 0.2 ± 0.09% siblings) were significantly depleted in JDM probands.

We also observed significantly differentially abundant ASVs in the saliva and supragingival swab samples from JDM probands, all of which were found in very low abundances. In the saliva, JDM probands were enriched with *Veillonella* (0.07 ± 0.06% JDM vs 0.05 ± 0.02% siblings) and *Prevotella shahii* (0.02 ± 0.008% JDM vs 0.009 ± 0.009% siblings). In contrast, *Prevotella melaninogenica* (0.005 ± 0.003% JDM vs 0.01 ± 0.01% siblings) was depleted in JDM proband saliva samples. In the supragingival swab samples, the most significantly enriched ASVs in JDM probands were *Neisseria oralis* (0.2 ± 0.07% JDM vs 0.09 ± 0.06% siblings), 2 unclassified species with high sequence identity to *Corynebacterium* (0.03 ± 0.01% JDM vs 0.02 ± 0.01% siblings), and *Prevotella* (0.04 ± 0.02% JDM vs 0.02 ± 0.01% siblings).

## Discussion

Our study examined oral and fecal microbiomes in JDM, a rare pediatric multi-systemic autoimmune disease of unclear etiology. We studied JDM probands in comparison to their unaffected family members, employing healthy siblings as the best possible controls to match for both age and environmental influences. The fact that JDM patients and their siblings were found to have such similar microbiomes proved advantageous: controlling (to some extent) for microbiome effects related to shared genetic and environmental contexts (including genetic determinants of the microbiome community structure, vertically transmitted microbiomes, shared dietary and cultural influences, and co-habitation) permitted us to identify microbiome features with a greater likelihood of being clinically pertinent to JDM. Despite JDM probands in our study having very mild disease, we identified several bacteria that were significantly enriched or depleted in JDM, and each was observed in low abundance, consistent with findings of prior studies investigating the microbiome in immune-mediated diseases^15^.

We observed significant enrichment of *Faecalibacterium* and *Ruminococcaceae* (which are both members of the *Lachnospiraceae* family) in the fecal microbiomes of children with JDM. *Faecalibacterium* are typically associated with anti-inflammatory properties, and while adult studies elucidate their impact on inflammation^16,17^, their immune-modulating effects in children are unknown. A systematic review of fecal microbiomes in pediatric IBD reported a decrease in *Faecalibacterium* compared to healthy controls within a majority of the 41 included studies^18^. Intriguingly, *Faecalibacterium* are also reported to be are enriched in the gut microbiomes of children with Crohn’s disease^19^. These mixed reports could be related to differences in the subclass of disease studied (ulcerative colitis vs Crohn’s disease) or strain-to-strain variability in *Faecalibacterium*. Altered representation of *Faecalibacterium* has also been reported in Sjögren’s syndrome^20^, RA^21^, and juvenile idiopathic arthritis (JIA)^22,23^. Fecal abundances of *Ruminococcus* species are greater in individuals with systemic lupus erythematosus, where it correlated with disease activity, and in patients with adult dermatomyositis (DM)^24^. These findings add to a body of evidence linking *Lachnospiraceae* bacterial species to immune-mediated diseases.

Additionally, we observed a significant enrichment of *Roseburia* and *Muribaculaceae* in the fecal microbiomes of children with JDM. *Roseburia* species have previously been associated with other autoimmune diseases such as type 1 diabetes^25^ and have the potential to activate autoantibody responses in vivo^26^. Levels of *Muribaculaceae* have previously been shown to positively correlate with disease activity as well as pro-inflammatory cytokines IL-17, TNF-α, and IFN-γ in RA patients^27^.

*Subdoligranulum*, on the other hand, was a depleted taxon, consistent with a previous study that reported lower *Subdoligranulum* in Crohn’s disease, suggesting it may be a putative probiotic in multiple contexts^28^. Indeed, *Subdoligranulum variabile* may be a potentially key member of the healthy human gut microbiome^29^. Furthermore, a recent study on juvenile idiopathic arthritis (JIA) in a small Swedish cohort indicated that *Subdoligranulum* abundances were significantly lower in the microbiomes of 1-year old children later diagnosed with JIA compared to controls, suggesting that microbiome differences may precede the onset of diagnosis^30^.

Interestingly, we observed significant decreases in 3 different *Streptococcus* sequences in the fecal samples of JDM probands, which contradicts previous reports of positive associations with autoimmune diseases, such as RA^21^, type 1 diabetes^25^, and Sjögren’s syndrome^20^. This discrepancy could be due to differences in the disease nature of JDM, the age of affected individuals, use of immunosuppressive medications, or in the *Streptococcus* species themselves. Antibodies reactive to an epitope in *S. pyogenes* has been reported in patients with JDM and were found to be cross-reactive with a muscle-specific myosin protein M5^31^. Whether the antibodies contribute to depletion of *Streptococcus* in the gut or are unrelated cannot be determined from the data available.

Different bacteria comprised JDM signatures of oral samples; in fact, few ASVs were found in both oral and stool samples, consistent with recent studies suggesting that oral bacteria do not colonize the distal gut^32,33^. In the anterior subgingival dental plaque, two *Fusobacterium* ASVs were significantly enriched in JDM probands, consistent with previous studies reporting increased abundance in RA^34^ and periodontal disease^35^, which has been linked to JDM^36^. Additionally, posterior subgingival dental plaque from JDM probands were enriched for *S. pneumonia*, which displays a plasma binding protein on its cell wall that may be immunogenic^37^. We also observed a significant enrichment of *Neisseriaceae* and *Laurotrpia* in JDM probands which have both been associated with RA^34^. Other notable enriched genera in JDM probands included *Campylobacter* and *Gemella* which have been associated with periodontal disease and JIA, respectively^38,39^. Conversely, we observed a loss of *Corynebacterium matruchotii* abundance in JDM probands, consistent with reports of lower levels in RA, JIA, and periodontal disease^40,41^. JDM probands also had a significant reduction in *Porphyromonas pasteri* (the most abundant and prevalent *Porphyromonas* species in healthy adults^42,43^) compared to their healthy siblings. *Porphyromonas* has been associated with RA in the subgingival plaque^34,40^. A recent report of Sjögren’s syndrome found higher abundance of *Porphyromonas pasteri* in the oral microbiome of healthy controls^44^. Though our findings in the saliva and supragingival swab samples indicated that the microbial differences between JDM probands and their healthy siblings are among low abundant bacteria, we still observed interesting differences. For instance, JDM probands were enriched with *Veillonella* and *Prevotella* species in their saliva. A recent report of RA patients suggested that these microbes could predispose patients to the development of this autoimmune disease in the early stages^45^, consistent with our observations. Additionally, the increased abundance of a *Neisseria* species in the supragingival swab samples of JDM probands corroborates similar findings in the anterior subgingival dental plaque. The observation that species from the same genera (e.g. *Streptococcus and Prevotella*) were both enriched and depleted in JDM probands suggests that microbial-disease associations may be species-specific. Interestingly, each of these species has been correlative with autoimmune and periodontal diseases in other studies^34,46^. Thus, analyses using higher-level taxonomic classifications may lose the granularity needed to determine disease associations with specific bacterial species. Future studies involving cultivation and characterization of these, and other novel bacterial isolates derived from JDM patient samples, may provide insights into pathogenesis. Our findings suggest that in depth microbial investigations of fecal and subgingival plaque samples (and perhaps less so salivary or supragingival swab samples) may be particularly revealing.

We found that the nuclear family unit had a very strong impact on oral and gut microbiomes, as expected. In our cohort, children had lower alpha diversity than adults, consistent with prior reports demonstrating increasing alpha diversity throughout childhood until a plateau in early adulthood^13,47^. Unweighted UniFrac distances of fecal samples revealed JDM probands were more similar to their siblings than to their parents and other probands. These observations suggest that the sibling relationship has a larger effect on gut microbiome similarity than does carrying a diagnosis of JDM, and the similarities among siblings are most pronounced with respect to less abundant taxa. As the youngest child recruited was 3.5 years, this observation is not confounded by the rapidly evolving developmental microbiome program that occurs in the first 3 years of life^13,47^. Together, these findings offer further support to the generally accepted notion that environmental exposures play major roles in microbiome assembly and establishment in childhood. Overall, we observed smaller distances between family members living in the same household compared to unrelated individuals—evidencing convergence of microbiomes of unrelated individuals attributable to living in the same household, particularly with respect to rarer microbes^48^. Oral microbiomes of children within a household (i.e., JDM probands and their siblings) were no more similar than the microbiomes of adults in the same household. This finding may reflect effects of kissing, common meals and similar dietary habits, direct sharing of utensils, etc., among family members, although these specific aspects of family life were not queried in our study.

Our study has several limitations. First, we lacked control families without any immune-mediated diseases. We attempted to overcome this limitation by analyzing published datasets and found evidence of familial dysbiosis. However, based on the available data and our current analysis, we are unable to draw conclusions regarding immunological effects of these familial microbiome differences. One compelling hypothesis is that interactions between one’s genetics and microbiome drive immune dysfunction a la the “ecological model of dysbiosis”^49^. While we were able to confirm that all NIH enrolled parents lived with their children, we were unable to confirm this for the SCH cohort. Given our small sample size, we were not able to do meaningful sub-analyses to demonstrate differences between known living with and living without a JDM proband. Second, our sample collection methods were designed solely for DNA-based profiling. Therefore, we could not culture bacterial isolates for direct testing of specific bacteria or bacterial metabolites in model systems of dysregulated immunity to study their functional impact. Third, our study was cross-sectional in design, a feature often inherent to studying a rare disease. Enrolled subjects largely had well-controlled or mildly active disease and were receiving immunosuppressive therapies, so we were unable to test for associations between microbial entities and disease activity. The study of Bae et al. did find a correlation of disease damage in patients with DM with lower microbial diversity^24^. We were unable to distinguish potential disease-driving bacteria from those whose representation was altered because of microenvironmental changes. An ideal future study would be a longitudinal multicenter study where we could sample the microbiome of new-onset JDM patients just prior to initiation of therapy, at timepoints during therapy, and upon achieving remission to assess for changes in the microbiome. Fourth, due to limitations in sample size and medication use history, we were unable to assess or control for the effects of specific drugs and drug classes. It is known that immunosuppressive drugs such as methotrexate^50^, oral corticosteroids (i.e., prednisone)^51^, and mycophenolate mofetil^52^ can alter the structure of bacterial communities. Despite these therapies, JDM probands and their siblings displayed high similarities in overall composition of oral and gut microbiomes.

Rare chronic pediatric diseases such as JDM are often challenging to diagnose and treat, and their diagnosis impacts entire family units. In this novel study of the oral and fecal microbiomes in JDM, in which we profiled JDM probands and their unaffected family members, we showed that family is a major influence on microbiome variability. This challenges the clinical paradigm of focusing on affected individuals and raises the prospect of whether practitioners ought to focus on family units in characterizing the microbiomes of patients. More broadly, our findings raise the question of whether microbiome-based therapies can ultimately succeed in treating individuals in isolation, or whether familial and social network units need to be addressed as a whole. In an analysis adjusting for microbiome differences attributable to family, we nevertheless found differences in several potentially immunomodulatory bacteria in patients with JDM. This study along with a recently-published study of the fecal microbiota in adult DM patients^24^ provides an initial microbial landscape to contextualize future studies. Further research will be needed to understand the role of these bacteria in disease pathogenesis and whether targeting them with microbiome-based interventions may be of clinical therapeutic value.

## Methods

### Human subjects

This prospective observational study was conducted in 2018–2019. Patients and their families were recruited from the Seattle Children’s Hospital (SCH) Rheumatology Clinic (protocol ID 1044) and the National Institutes of Health (NIH) Clinical Center (as part of the NIEHS Twins and Siblings Discordant for Systemic Rheumatic Diseases study; protocol ID 03-E-0099) using institutional review board-approved research protocols for informed consent (obtained from a parent and/or legal guardian in the case of minors), medical record review, clinical sample collection, and use of samples. All methods were performed in accordance with the relevant guidelines and regulations. JDM probands were children 2–18 years of age who met probable or definite criteria for JDM (as defined by Bohan and Peter criteria for myositis^53,54^). Siblings in both cohorts had no underlying autoimmune disease and had an age difference of ≤ 5 years, except for 2 siblings from the SCH cohort (JDM0019R1 and JDM0020R1) who were within 10 years of age. Exclusion criteria for JDM probands were other co-existing immune-mediated diseases; for siblings, any autoimmune or immune-mediated disease diagnoses; and for all participants, antibiotics received within the past 3 months. A total of 28 children with JDM (probands), 27 unaffected siblings (representing 26 families; in one family, a JDM proband had 2 unaffected siblings enrolled), 26 mothers, and 17 fathers were enrolled, resulting in a collection of 33 fecal, 95 saliva, 94 supragingival swab, 19 anterior subgingival dental plaque, and 19 posterior subgingival dental plaque samples. Fecal samples were collected using DNA Genotek OMNIgene Gut (OMR-200). Oral samples were collected in two ways: (i) anterior and posterior subgingival dental plaque samples were collected with sterilized Gracey currettes from 3 anterior teeth and 3 posterior teeth, and (ii) supragingival swabs were collected with OMNIgene Oral (OM-505) kits. All samples were placed in the same buffer for stabilization of nucleic acids and stored at -80°C until use. Medication use and laboratory parameters of myositis disease activity were extracted from patient charts (SCH) or prospectively collected at study visits (NIH). Disease duration was calculated using information collected from patients via case report form (CRF) and subsequently verified by chart review. Myositis disease activity was assessed using two validated metrics: Manual Muscle Testing 8 (MMT-8), with bilateral testing of the muscle groups (score 0–150) and the Cutaneous Dermatomyositis Disease Area and Severity Index (CDASI)^55,56^.

### Genomic DNA extraction

Samples (250 µL per stool sample, 1 mL per saliva sample, or 1 mL per plaque sample) was homogenized at 25–30 Hz for 10 min using a Qiagen TissueLyser II. Genomic DNA was then extracted and purified using either the Qiagen PowerFecal Pro DNA Kit (fecal samples; as per manufacturer’s instructions) or the Qiagen QIAamp DNA Microbiome Kit (saliva and plaque samples; host DNA depletion step skipped to minimize concomitant loss of bacterial DNA). Genomic DNA was quantified using a Qubit fluorometer.

### 16S rRNA sequencing and analysis

Hypervariable region 4 (V4) of 16S ribosomal RNA genes present in samples was PCR amplified with 515F (5′-GTGCCAGCMGCCGCGGTAA-3′) and 806R (5′-GGACTACNVGGGTWTCTAAT-3′) primers using Taq high-fidelity polymerase (Invitrogen) in samples containing normalized quantities of genomic DNA. Sample-specific barcoded adapters were used to prepare and pool sequencing libraries as previously described^57–59^. To minimize batch effects and bias, we (i) employed pseudo-randomization during library preparation, (ii) minimized the number of sequencing runs (2 total runs: all stool samples in 1 run and all oral samples in the other run), (iii) used an automated liquid handler for multiple steps during library preps, and (iv) applied Conditional Quantile Regression during analysis remove microbiome batch effects^14^. Researchers generating the sequencing data were blinded to clinical metadata associated with samples. Multiplexed sequencing was performed on the Illumina MiniSeq platform (150 nucleotide paired-end reads; 20% PhiX). 12 samples (8 saliva from 4 patients, 3 siblings, and 1 father; 4 supragingival swabs from 2 patients and 2 siblings) were excluded due to low numbers of remaining high-quality reads, likely attributable to insufficient amounts of bacterial DNA captured in the original sample. Quality filtering, merging of mate pairs, and determination of amplicon sequence variants (ASVs)^60^ was performed using DADA2^61^ via QIIME2^62^. Sequencing depth of retained samples was 131,263 ± 7141 (mean ± SEM) high-quality reads per sample. ASVs with a relative abundance < 0.001% and those present in only 1 sample throughout the entire dataset were removed prior to further analysis. Taxonomic classifications were performed in QIIME2 using a classifier trained on 515F-806R sequences from the Silva database^62^. Selected sequences classified as “unannotated bacteria” were subjected to an NCBI BLAST search against the *nr* database using a sequence identity threshold of > 98%. Diversity analyses were performed using QIIME2 and the R package *phyloseq* (version 1.30.0). Alpha diversity calculations were performed after rarefaction to 6,100 reads per sample. Beta diversity calculations were made using UniFrac, a phylogenetic metric that calculates the proportion of a phylogenetic tree shared by two samples^63^, both with and without rarefaction of sequencing data; the latter is recommended by Holmes, Willis, and colleagues^64,65^ and is reported here.

### Myositis autoantibodies

Sera were tested for myositis-specific (MSA) and myositis-associated autoantibodies (MAA) using validated immunoprecipitation (IP) and IP-immunoblotting methods at the Oklahoma Medical Research Foundation laboratory^66^.

### Downloaded datasets

Datasets were downloaded from using accession numbers listed in the original publications^11–13^. To control for batch effects, we applied ConQuR^14^, which uses a two-part quantile regression model to perform batch correction.

### Statistics

Statistical analysis was performed in R (version 4.0.3) using the specific tests named in the text. To identify significantly differentially abundant bacterial taxa while adjusting for family as a covariate of interest, we used *corncob*, an R package that leverages a beta-binomial regression model and uses the Wald test to calculate significance^67^. In our analysis, we also adjusted for sequencing depth, a potential source of measurement noise^68^. The effects of covariates on UniFrac distances were calculated using permutational multivariate analysis of variance (PERMANOVA). In follow-up analyses, specific comparisons were performed using a Student’s *t*-test. A *p*-value < 0.05 was considered statistically significant. Plots were generated in R using *ggplot2* (version 3.3.5) and *phyloseq* packages (version 1.30.0) and then assembled in Adobe Illustrator (2022 version).

### Ethics approval and consent to participate

Informed written consent was obtained from and documented for all study participants. The research methods used were approved by the Institutional Review Boards at Seattle Children’s Hospital (protocol ID 1044) and at the National Institutes of Health (protocol ID 03-E-0099).

### Supplementary Information

Supplementary Table 1.

Supplementary Table 2.

Supplementary Figure 1.

Supplementary Figure 2.

Supplementary Legends.

## Data Availability

The datasets generated and analyzed during the current study will be made available in the ENA repositories PRJEB50544, PRJEB75379, PRJEB75444, PRJEB75445, and PRJEB75446 at the time of publication.

## Abbreviations

JDM: Juvenile dermatomyositis
NIDCR: National Institute of Dental and Craniofacial Research
NIH: National Institutes of Health
NIEHS: National Institute of Environmental Health Sciences
SCH: Seattle Children’s Hospital
MMT: Manual Muscle Testing
CDASI: Cutaneous Dermatomyositis Disease Area and Severity Index
MSA: Myositis-specific autoantibodies
MAA: Myositis-associated autoantibodies
ANA: Anti-nuclear antibody
IP: Immunoprecipitation
PCoA: Principal coordinates analysis
PERMANOVA: Permutational multivariate analysis of variance
IBD: Inflammatory bowel disease
ASV: Amplicon Sequence Variant
RA: Rheumatoid arthritis
JIA: Juvenile idiopathic arthritis
