# Supplementary figures and images for: Familial clustering of dysbiotic oral and fecal microbiomes in juvenile dermatomyositis
Source: Sci Rep. 2024 Jul 12;14:16158. doi: 10.1038/s41598-024-60225-0 (PMC11245510; doi:10.1038/s41598-024-60225-0)

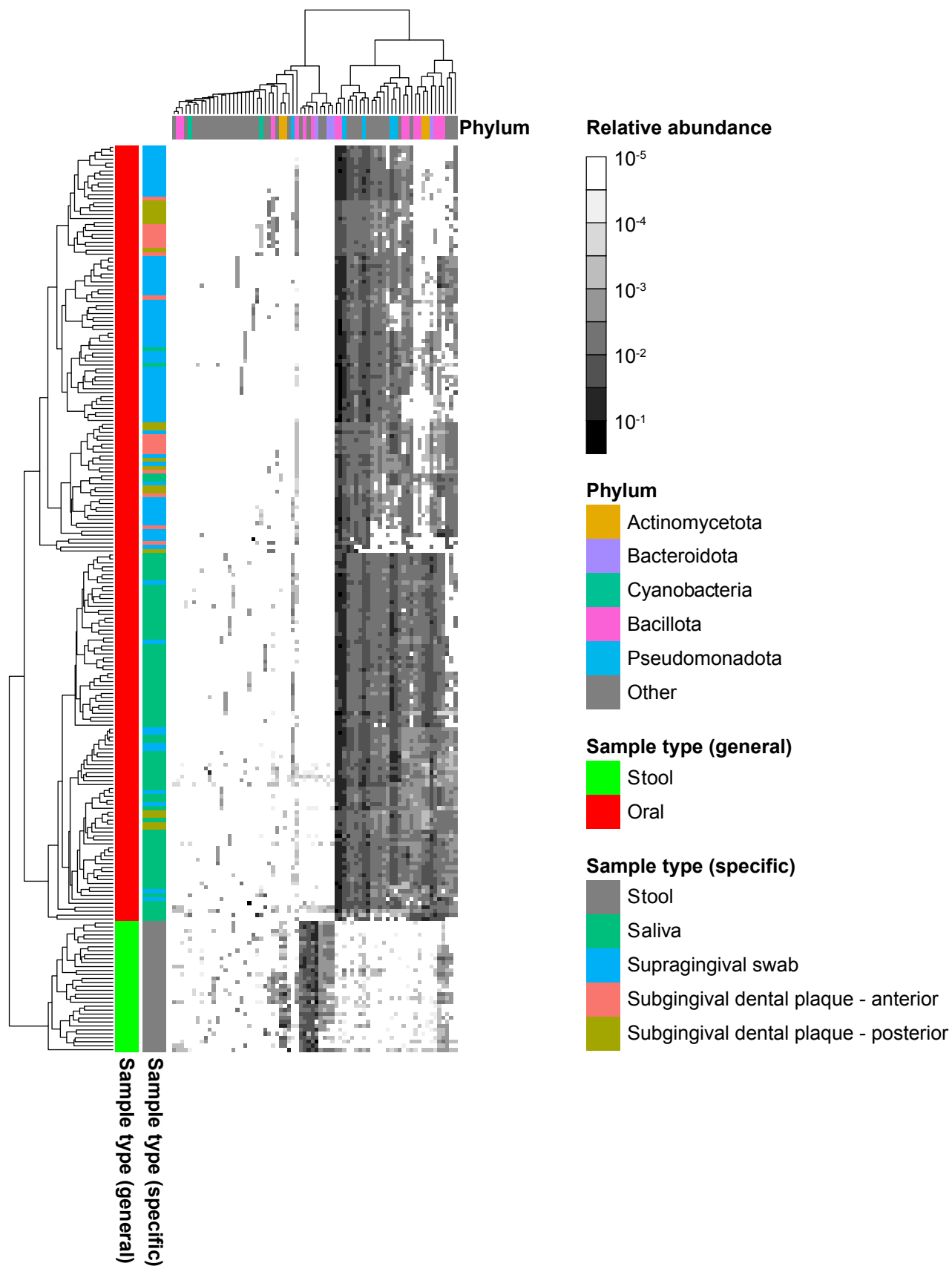

Supplement: Supplementary file 3 — Supplementary Figure 1. [file 41598_2024_60225_MOESM3_ESM.pdf]

**A**

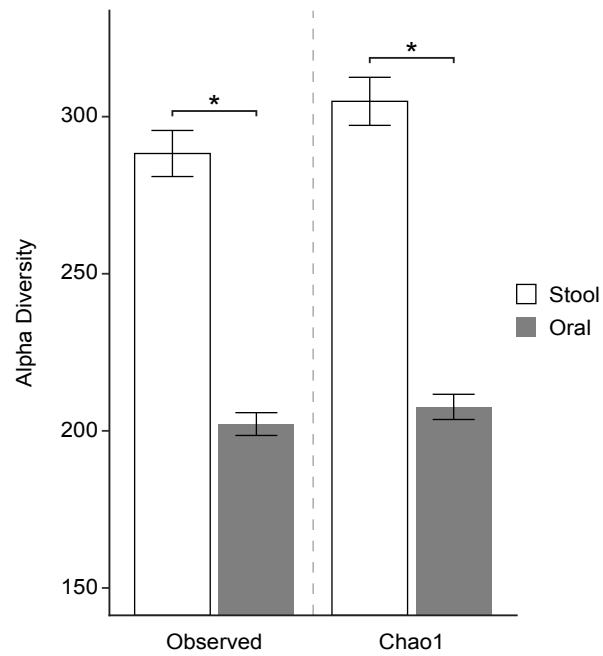

**B**

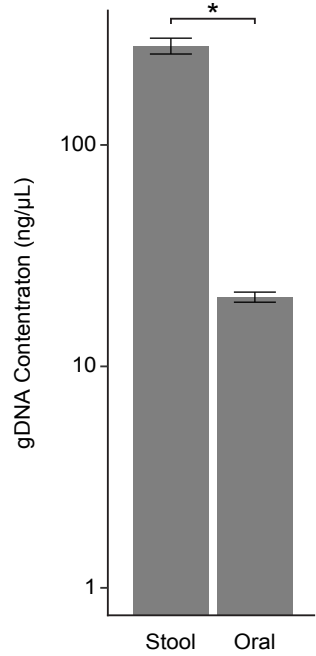

Supplement: Supplementary file 4 — Supplementary Figure 2. [file 41598_2024_60225_MOESM4_ESM.pdf]
