# Supplementary material for: Familial clustering of dysbiotic oral and fecal microbiomes in juvenile dermatomyositis
Source: Sci Rep. 2024 Jul 12;14:16158. doi: 10.1038/s41598-024-60225-0 (PMC11245510; doi:10.1038/s41598-024-60225-0)
Supplement: Supplementary file 5 — Supplementary Legends. [file 41598_2024_60225_MOESM5_ESM.docx]

# SUPPLEMENTARY FIGURE LEGENDS

**Figure S1.** Characteristics of ASVs detected in both oral and fecal microbiomes. The 72 ASVs that were shared between fecal and oral microbiomes comprised of diverse phyla, constituted <1% of total relative abundance in samples (on average), and clustered predominantly by sample type (i.e., oral versus fecal) rather than by individual or family. Each column is a different ASV, and the heatmap represents the relative abundance in each sample.

**Figure S2. (A)** Alpha diversity, as calculated using observed species and Chao1 metrics, and **(B)** genomic DNA concentrations of fecal vs oral samples.

# SUPPLEMENTARY TABLE LEGENDS

**Table S1.** Catalog of samples collected at SCH and NIH as part of the present study. Study participants from both sites were ultimately assigned a person ID of the same format ("JDM" followed by 4-digit family ID followed by relationship code; "A" = proband, "M" = mother, "F" = father, "R" = sibling). “X” indicates that a sample was collected. Dark gray filled boxes indicate samples that were not collected. Light gray boxes indicate samples that were collected but excluded from the final analysis due to low genomic DNA yield and/or poor-quality sequencing data.

**Table S2.** DNA sequencing datasets generated in this study and downloaded from others' studies that were used in the analyses presented herein. **(A)** Bacterial V4-16S rRNA sequencing datasets generated from samples collected at SCH and NIH. **(B)** All ASVs seen in our dataset and their corresponding taxonomic classifications. **(C)** Downloaded bacterial V4-16S rRNA sequencing data.
